# Novel Ataxia Telangiectasia and Rad3-Related Protein (ATR) Phenotype: Marfanoid Appearance, Generalized Hypermobility, Gait Imbalance, and Poor Wound Healing: A Case Report

**DOI:** 10.7759/cureus.100178

**Published:** 2025-12-27

**Authors:** Zoie A Sadler, Mark D Unger

**Affiliations:** 1 Osteopathic Manipulative Medicine &amp; Osteopathic Principles and Practices, Liberty University College of Osteopathic Medicine, Lynchburg, USA

**Keywords:** atr related disorder, connective tissue disorder, genetic testing, physical exam, rheumatology

## Abstract

Connective tissue disorders present to the emergency department with a broad spectrum of symptoms and signs, often leading to delayed diagnosis and missed opportunities to prevent fatal complications later in life. We report a case of a 54-year-old woman with an extensive history of multiple repeated medical evaluations for nonspecific complaints, including hypermobility, poor wound healing, and impaired balance, without a definitive diagnosis for six years. Eventually, the patient underwent whole exome sequencing (WES). WES identified a heterozygous variant in the ataxia telangiectasia and Rad3-related protein (ATR) gene, NM_001184.4(ATR):c.1464G>T (p.Glu488Asp), previously classified as a variant of uncertain significance based on only one prior report. The present case may represent a novel manifestation of ATR-related disorders, suggesting considerable overlap with hereditary connective tissue disorders. Rapid bedside assessment for connective tissue abnormalities in patients presenting with nonspecific symptom complexes could facilitate earlier identification of appropriate candidates for genetic testing.

## Introduction

Hereditary connective tissue disorders (HCTD) affect the normal structure of connective tissue, a vital component of bodily support and organ function [1]. Key systems commonly affected by HCTD include the cardiovascular, gastrointestinal, and musculoskeletal systems; however, all organ systems can potentially be affected [1]. Due to the ubiquitous role of connective tissue in health, many patients with an underlying HCTD present with nonspecific symptom complexes [1]. 

Diagnosis of HCTD often requires the clinician to connect a vague constellation of symptoms and signs with a family history of positive genetic test results, which relies on an expanding library of genetic markers [1]. Due to the likely incomplete library of genetic markers and nonspecific symptoms, HCTD often remains undiagnosed for years [2]. In addition to poorly defined diagnostic parameters, some HCTDs, such as Marfan syndrome, can occur without a family history due to sporadic gene mutations, which limits the diagnostic utility of a negative family history [3]. 

Undiagnosed or misdiagnosed cases can lead to severe complications, such as aortic aneurysms in Marfan syndrome [4], interstitial lung disease in scleroderma [5], and vascular fragility in Ehlers-Danlos syndrome (EDS) [6]. Diagnosis of EDS requires a high index of suspicion, especially in patients who deny a history of poor wound healing, easy bruising, joint dislocations, or hypermobility, which is assessed at the bedside via measurement of the Beighton score [7]. In this context, we report the second known case of a specific ataxia telangiectasia and Rad3-related protein (ATR) variant, NM_001184.4(ATR):c.1464G>T (p.Glu488Asp), in a patient with Marfanoid appearance, generalized hypermobility, gait imbalance, and poor wound healing. The phenotypic spectrum of ATR mutations remains incompletely characterized; however, some previously described cases involving ATR mutations demonstrate varied musculoskeletal anomalies [8]. While the overall prevalence of ATR variants is not currently reported, the two with musculoskeletal abnormalities, Seckel syndrome and Familial Cutaneous Telangiectasia and Cancer Syndrome, have a reported incidence of 1: 10,000 children and a prevalence of <1:1,000,000, respectively [9,10]. This case report illustrates the utility of bedside assessment for connective tissue abnormalities in patients complaining of nonspecific symptoms to help identify appropriate candidates for genetic testing earlier in the disease course.

## Case presentation

A 54-year-old Caucasian female presented on multiple occasions to the emergency department (ED), urgent care, and primary care office with repeated complaints. Repeat complaints included generalized abdominal pain, migratory arthralgias affecting small, medium, and large joints, easy bruising, and poor wound healing. The patient also frequently complained of chronic fatigue, unintentional weight fluctuations of up to twenty pounds, fever of unknown origin, cold intolerance, weakness, and falls due to balance impairment. No definitive diagnosis or satisfactory treatment was achieved over the course of four years. During this time, laboratory values were consistently within reference ranges, including complete blood counts, comprehensive metabolic profiles, thyroid function tests, vitamin D, vitamin B12, vitamin B9, copper, and hemoglobin A1C. Viral testing was negative for human immunodeficiency virus, cytomegalovirus, herpes simplex virus 1 and 2, and Epstein-Barr virus. The patient had no family history of intellectual disability, birth defects, obstetrical complications, or genetic disorders. As a child, the patient endorsed clumsiness, frequent injuries, and recurrent upper respiratory illnesses. Significant medical history as an adult included palpitations, malignant melanoma, insomnia, and Raynaud phenomenon.

After four years of failed medical care, the patient presented to the authors’ Osteopathic Neuromusculoskeletal Medicine clinic seeking symptomatic management of chronic pain affecting the bilateral neck, shoulders, hips, upper extremities, and lower extremities. The patient complained of weakness and dysesthesia affecting the bilateral upper and lower extremities. Physical exam was significant for Beighton score of 7/9, arachnodactyly, arm span to height ratio of 1.05, pectus excavatum, pes planus, scoliosis, wrist and thumb signs, and myopia. The Marfan systemic score was eight. Neurologic exam revealed 1+/4 deep tendon reflexes at the biceps, brachioradialis, triceps, patella, and Achilles bilaterally. Strength was 4/5 with hip flexion, knee extension, great toe extension, and ankle plantarflexion bilaterally. Sensation was decreased to light touch, pinprick, temperature, and vibration in the bilateral upper extremities distal to the wrists and in both legs distal to the mid-calves. Tandem gait was unsteady. The Romberg test revealed increased sway. The patient elected for symptomatic management of widespread chronic pain through a combination of osteopathic manipulative treatment to address recurrent somatic dysfunction and medicinal management, including gabapentin and tizanidine.

**Figure 1:**
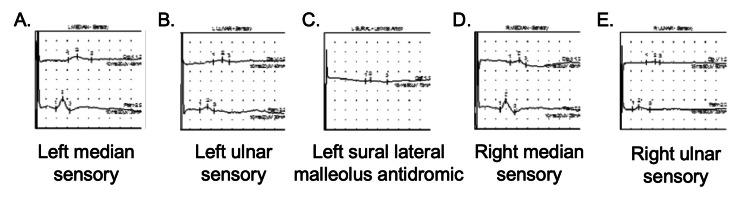
Waveform of sensory component of EMG/NCS demonstrating peripheral neuropathy with multi-nerve diminished segment response A. Left median nerve B. Left ulnar nerve C. Left sural lateral malleolus antidromic D. Right median nerve E. Right ulnar nerve

The patient experienced inconsistent improvement in pain over the following two years. The severity of both her gait instability and dysesthesias increased, affecting her ability to perform fine motor skills. She developed tension headaches, migraine with aura, and unintentional weight loss of 43 pounds over nine months. Additional multidisciplinary evaluation was sought, including orthopedics, neurology, cardiology, hematology/oncology, and medical genetics. Noted findings from additional diagnostic workup included transesophageal echocardiography demonstrating aortic root dilation of 3.8 cm and cardiac rhythm monitoring demonstrating atrial fibrillation. Multiple advanced imaging studies were unremarkable, including magnetic resonance imaging of the brain with and without intravenous contrast and computed tomography of the neck, thorax, abdomen, and pelvis with and without intravenous contrast. Electromyography/nerve conduction study demonstrated widespread sensory peripheral neuropathy affecting the bilateral upper and lower extremities (Table 1, Figure 1, Appendices 1-5). Sensory component of electromyography (EMG)/nerve conduction study (NCS) demonstrating electrophysiological evidence of widespread sensory peripheral neuropathy.

**Table 1:** Sensory component of electromyography (EMG)/nerve conduction study (NCS) demonstrating electrophysiological evidence of widespread sensory peripheral neuropathy. L - left, R - right, Dig - digit, Ref - reference electrode, Lat Mall - lateral malleolus, Antidr - antidromic, Rec - recording, Peak Lat - peak latency, ms - millisecond, NP Amp - negative peak amplitude, μV - microvolts, SPAR% - sensory potential amplitude ratio, cm - centimeters, m/s - meters per second

Nerve/Sites	L MEDIAN - Sensory				R. Median - Sensory				L. Ulnar - Sensory				R. Ulnar - Sensory				L. SURAL- Lat Mall Antidr	
	Dig II	Ref.	Palm	Ref.	Dig II	Ref.	Palm	Ref.	Dig V	Ref.	Palm	Ref.	Dig V	Ref.	Palm	Ref.	Calf	Ref.
Rec Site	Wrist		Wrist		Wrist		Wrist		Wrist		Wrist		Wrist		Wrist		Lat mall	
Peak Lat ms	3.90	3.50	2.55	2.20	4.29	3.50	2.95	2.20	4.19	2.90	2.65	2.20	4.90	2.90	2.60	2.20	6.50	4.20
NP Amp μV	7.5	10	19.8	40.0	5.6	10.0	16.7	40.0	4.5	5.0	6.8	20.0	1.5	5.0	13.3	20.0	5.6	5.0
PP Amp μV	6.7		23.7		10.2		23.4		5.2		9.1		4.5		14.8		9.9	
SPAR %	100	50	100		75	50	84.4		100	50	51.2		33.6	50	100			50
Segments	Dig II	Ref.	Palm	Ref.	Dig II	Ref.	Palm	Ref.	Dig V	Ref.	Palm	Ref.	Dig V	Ref.	Palm	Ref.	Calf	Ref.
Distance cm	13		8		13		8		11		8		11		8		14	
Velocity m/s	42.6		42.1		38.3		33.3		33.8		41.0		29.7		45.7		24.1	

Repeat complete blood counts and comprehensive metabolic profiles remained within reference ranges, and protein electrophoresis identified an incidental M spike of 0.2 g/dL, leading to a diagnosis of monoclonal gammopathy of undetermined significance. Whole exome sequencing (WES), which included approximately 20,000 genes, demonstrated a single heterozygous mutation in the ATR gene: NM_001184.4(ATR):c.1464G>T (p.Glu488Asp).

## Discussion

This patient’s medical journey lasted over six years before WES was performed, and identified a single heterozygous mutation. The patient’s workup involved multiple presentations to the ED, urgent care, and primary care setting,s along with numerous subspecialty referrals for seemingly unrelated symptoms and signs. This case underscores the importance of maintaining a broad differential diagnosis that includes an expanding landscape of genetic variants that could underlie dangerous and life-threatening complications. Nathanael Turner highlights this perspective, writing that primary care represents both “the first point of contact and the last line of defense” for our patients [11]. This burden is faced by ED providers, who may be the initial contact for many patients who present with nonspecific complaints due to a yet-to-be-recognized condition [12]. Earlier consideration of connective tissue disorders in our patient might have expedited genetic testing, potentially averting delays in care.

For example, rheumatic complaints account for up to 8% of ED visits, which underscores the importance of assessment tools, such as the Beighton score and the Marfan systemic index, in patients with a history of frequent musculoskeletal injuries or generalized pain [13]. The current case provides context because the patient presented to the ED, urgent care, and primary care settings on multiple occasions, complaining of partial symptoms and signs in the presence of a single heterozygous mutation in the ATR gene. Perhaps the most significant and underappreciated clinical sign was the patient’s Beighton score of 7/9 that indicated generalized hypermobility, equating to 3 points above her age-specific threshold [14]. Assessing the Beighton score in acute settings is feasible due to the speed with which it can be performed and could improve diagnostic accuracy for complex cases such as this patient [7, 15].

The present case history mostly aligns with HCTD, including EDS or Marfan syndrome, despite the absence of an associated gene variant. On the other hand, the patient’s sole gene variant was localized in the coding region of ATR. ATR-related disorders are rare [16] and relatively understudied. Table 2 provides a summary comparison between the patient presentation and previously described ATR-related conditions, including Familial Cutaneous Telangiectasia and Cancer Syndrome, a heterozygous manifestation of ATR-related disorders characterized by ataxia, telangiectasias, immune dysfunction, and lymphoma predisposition, typically presenting early on in life [8]. The patient’s age and partial overlap with both ATR-related disorders and HCTD suggest a novel phenotype that may support reclassification of the NM_001184.4(ATR):c.1464G>T (p.Glu488Asp) variant [17].

**Table 2:** Summary comparison of genotype-phenotype relationships between the patient and classic ataxia telangiectasia and Rad3-related protein (ATR)-related disorders.

Genetic and Phenotypic Factors	Known Homozygous ATR Mutations	Known Heterozygous ATR Mutations	Patient	Summary Comparison of Patient Presentation with Existing Literature
Inheritance and Onset	Seckel Syndrome is autosomal recessive. Hallmarks include congenital/early childhood onset features and intrauterine growth issues [18].	Characterized through the study of autosomal dominant cancer syndromes. Typically, adult-onset, often cancer-focused presentation without early developmental signs [8].	Heterozygous and adult-onset: symptoms escalating from about age 50 with childhood clumsiness but no severe early issues.	Patient demonstrated a later onset and broader multisystem involvement beyond cancer risk.
Growth and Development	Severe microcephaly, dwarfism, intrauterine/postnatal growth retardation, intellectual disability, developmental delay [8].	Typically, normal growth without intellectual disability may include systemic features like premature aging in animal models [19].	Normal growth with arm span/height ratio 1.05 and no microcephaly or dwarfism, intellectual disability.	The patient lacked hallmarks of Seckel Syndrome.
Neurological Features	Microcephaly with intellectual disability. Possible defects in cellular DNA damage response [20].	Rare neurological involvement, though some evidence for age-related neurodegeneration in models.	Peripheral sensory neuropathy, gait instability, intractable headaches, and visual disturbances. Normal brain MRI. Abnormal electromyogram/nerve conduction study.	Patient demonstrated adult-onset neuropathy and balance issues absent in Seckel Syndrome, exhibiting more sensory-motor disturbances than cognitive or central defects in known heterozygous cases.
Musculoskeletal and Connective Tissue	Bird-like facial features and skeletal abnormalities, such as disproportionate short stature [8].	Minimal or largely undocumented.	Hypermobility, arachnodactyly, migratory arthralgias, chronic pain in neck/shoulders/hips/extremities, poor wound healing, protracted bruising.	Patient exhibited Marfan-like connective tissue features not reported in ATR disorders, a stark contrast with skeletal dwarfism in Seckel Syndrome.
Cardiovascular	Rare reports, but likely not a core feature.	Cardiac issues in some reports, but aortic dilation was not noted.	Aortic root dilation, atrial fibrillation, palpitations, and Raynaud’s phenomenon.	Patient exhibited non-aneurysmal vascular dilation and arrhythmia, unlike minimal cardiac involvement in known ATR phenotypes.
Systemic Features and Laboratory Findings	Normal routine labs, DNA repair defects at the cellular level, but no evidence of cancer predisposition [8,20].	Increased cancer risk (e.g., oropharyngeal, lung adenocarcinoma, melanoma) and systemic manifestations like weight loss, fatigue [8].	Normal routine labs, chronic fatigue, unintentional weight loss, fever of unknown origin, cold intolerance, abdominal pain, insomnia, M spike, history of malignant melanoma, recurrent upper respiratory illnesses in childhood.	Patient had increased cancer risk (melanoma) and systemic manifestations (weight loss, fatigue), like heterozygous cases, but patient presentation was unique for immune/hematologic features, gastrointestinal issues, and poor healing.

## Conclusions

We report a novel phenotype for the ATR-related disorders in a patient found to have a single heterozygous mutation: NM_001184.4(ATR):c.1464G>T (p.Glu488Asp). The patient exhibited Marfanoid appearance, generalized hypermobility, gait imbalance, and poor wound healing among other nonspecific symptoms and signs. The patient faced a six-year delay before WES was performed. Her history of repeat presentations to the ED, urgent care, and primary care settings for nonspecific complaints without diagnosis underscores the importance of incorporating bedside assessment tools like the Beighton score to recognize suitable candidates for genetic testing. Expedited diagnosis of HCTD could enable timely preventative management of disease-related complications such as vascular fragility or malignancy. As the genetic library continues to expand, uncommon variants may suggest new relationships between distinct conditions, such as ATR-related disorders and HCTD. Limitations of this study include the paucity of preexisting literature on ATR-related disorders for comparison. The broad symptomology of ATR-related disorders makes it difficult to understand the pathophysiological role of the patient’s ATR variant. As more patients are identified through genetic sequencing, it will be possible to better correlate a patient’s genotype and the corresponding phenotype.

## Figures and Tables

**Figure 2:**
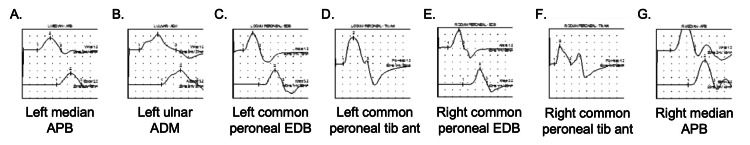
Waveform of motor component of EMG/NCS demonstrating normal to minimally deficient voluntary response without fasciculations A. Left median anterior pollicis brevis (APB) B. Left ulnar adductor digiti minimi (ADM) C. Left common peroneal extensor digitorum brevis (EDB) D. Left common peroneal tibialis (tib) anterior (ant) E. Right common peroneal EDB F. Right common peroneal tib ant G. Right median APB APB - anterior pollicis brevis, ADM - adductor digiti minimi, EDB - Extensor digitorum brevis, tib ant - tibialis anterior,

**Figure 3:**
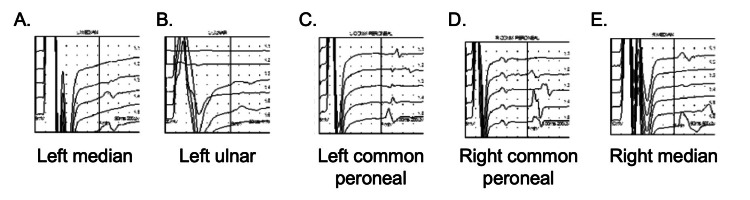
Waveform of F wave component of EMG/NCS demonstrating normal minimum F-wave latency. A. Left median B. Left ulnar C. Left common peroneal D. Right common peroneal E. Right median

## Appendices

Appendix 1 

**Table 3:** Motor component of electromyography (EMG)/nerve conduction study (NCS) within normal range. L - left, R - right, A. - anterior, COMM - common, APB - anterior pollicis brevis, ADM - adductor digiti minimi, EDB - Extensor digitorum brevis, Tib Ant - Tibialis Anterior, Fib - fibular, Ref - reference electrode, Rec - recording, Lat - latency, Amp - amplitude, ms - millisecond, Rel Amp % - relative amplitude percentage, SPAR% sensory potential amplitude ratio, Dist. - Distance, cm - centimeters, m/s - meters per second

Nerve/Sites	L MEDIAN - APB				R MEDIAN - APB				L ULNAR - ADM				L COMM PERONEAL - EDB				R COMM PERONEAL - EDB				L COMM PERONEAL - Tib Ant		R COMM PERONEAL - Tib Ant	
	Wrist	Ref.	Elbow	Ref.	Wrist	Ref.	Elbow	Ref.	Wrist	Ref.	A. Elbow	Ref.	Ankle	Ref.	Knee	Ref.	Ankle	Ref.	Knee	Ref.	Fib Head	Ref.	Fib Head	Ref.
Rec. Site	APB		APB		APB		APB		ADM		ADM		EDB		EDB		EDB		EDB		Tib Ant		Tib Ant	
Lat ms	4.05	4.40	9.15		3.70	4.40	8.45		2.95	3.50	8.90		5.35	5.70	13.80		5.55	5.70	13.55		5.20		3.50	
Amp mV	8.5	4.2	8.2	4.2	7.7	4.2	7.1	4.2	11.2	5.6	10.1	5.6	2.5	2.8	2.1	2.2	2.6	2.8	2.2	2.2	3.8		2.6	
Rel Amp %	100		95.9		100		92.3		100		89.7		100		81.3		100		87.5		100		100	
SPAR %	100	50	100		90	50	86.7			50			99	50	91.9		100	50	100		100	50	69	50
Segments	Wrist - APB	Ref.	Elbow-Wrist	Ref.	Wrist-APB	Ref.	Elbow-Wrist	Ref.	Wrist-ADM	Ref.	A. Elbow-Wrist	Ref.	Ankle-EDB	Ref.	Knee-Ankle	Ref.	Ankle-EDB	Ref.	Knee-Ankle	Ref.	Fib Head - Tib Ant	Ref.	Fib Head- Tib Ant	Ref.
Dist. cm	6.5		26		6.5		27		6.5		31		7.5		46		7.5		44					
Velocity m/s			51.0	49.0			56.8	49.0			52.1	49.0			54.4	41.0			55.0	41.0				

Appendix 2 

Appendix 3 

**Table 4:** F wave component of electromyography (EMG)/nerve conduction study (NCS) within normal range. L - left, R - right, COMM - common, Ref - reference electrode, Fmin - minimum F-wave latency, ms - milliseconds

Nerve	Fmin ms
L MEDIAN	30.75
REF	31.00
L ULNAR	31.20
REF	32.00
L COMM PERONEAL	59.00
REF	60.00
R COMM PERONEAL	59.35
REF	60.00
R MEDIAN	30.35
REF	31.00

Appendix 4 

Appendix 5 

**Table 5:** Summary table of electromyography (EMG)/nerve conduction study (NCS) L - left, R - right, D Inteross - Dorsal interosseous, Tib - Tibialis, Vast - vastus, MUAP - motor unit action potential, IA - insertional activity, Fib - fibrillation potentials, PSW - positive sharp wave, Fasc - fasciculation, H.F. - High Frequency, Amp - amplitude, Dur. - duration, PPP - polyphasic potentials, N - normal

EMG Summary Table									
	Spontaneous					MUAP			Recruitment
	IA	Fib	PSW	Fase	H.F.	Amp	Dur.	PPP	Pattern
R. FIRST D INTEROSS	N	None	None	None	N	N	N	N	N
R. DELTOID	N	None	None	None	N	N	N	N	N
R. TIB ANTERIOR	N	None	None	None	N	N	N	N	N
R. VAST LATERALIS	N	None	None	None	N	N	N	N	N
L. FIRST D INTEROSS	N	None	None	None	N	N	N	N	N
L. DELTOID	N	None	None	None	N	N	N	N	N
L. TIB ANTERIOR	N	None	None	None	N	N	N	N	N
L. VAST LATERALIS	N	None	None	None	N	N	N	N	N
